# RetroRules 2026: an expanded database combining biochemical and organic reaction templates for pathway discovery

**DOI:** 10.1093/nar/gkaf1261

**Published:** 2025-12-08

**Authors:** Thomas Duigou, Philippe Meyer, Jean-Loup Faulon

**Affiliations:** Micalis Institute, INRAE, AgroParisTech, Université Paris-Saclay, 78350 Jouy-en-Josas, France; Micalis Institute, INRAE, AgroParisTech, Université Paris-Saclay, 78350 Jouy-en-Josas, France; Micalis Institute, INRAE, AgroParisTech, Université Paris-Saclay, 78350 Jouy-en-Josas, France

## Abstract

RetroRules (https://retrorules.org) is an open resource of reaction templates, which are generic reaction representations that describe the atomic transformations underlying biochemical reactions. These templates are key to supporting metabolic pathway discovery, reaction prediction, and enzyme engineering. The 2026 release updates biochemical sources (MetaNetX, Rhea) and newly integrates organic chemistry reactions (USPTO), extending the scope of the database beyond enzymatic systems. The template encoding has been simplified by using implicit hydrogens and minimal atomic descriptors, resulting in faster and more compact representations. Radius range now spans 0–10, allowing finer control of reaction specificity. In addition, mass-imbalanced reactions are included, expanding the coverage of biochemically relevant transformations. Reaction mapping now relies on the transformer-based tool RXNMapper, improving accuracy. RetroRules 2026 comprises 1 174 216 templates derived from 92 698 reactions, covering 5796 fourth-level EC numbers. A redesigned website, updated Online Template Generator, and OpenAPI-defined API enable multi-criteria exploration (dataset, radius, and EC number), visualization, and data export in multiple formats. Sequence annotations from UniProt were refreshed and summarized as a normalized sequence-support score for ranking. Together, these updates establish RetroRules as a cross-domain resource bridging biochemistry and organic chemistry, offering broader coverage, controllable specificity, and enhanced usability for high-throughput pathway design, reaction prediction, and enzyme engineering.

## Graphical Abstract

**Graphical Abstract:**
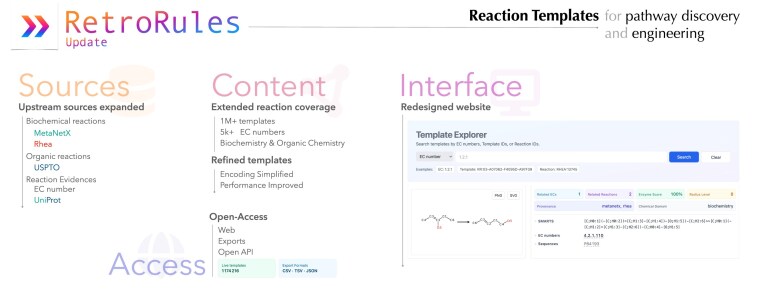

## Introduction

Chemical reactions can be abstracted into reusable patterns, called reaction templates [1], that capture the atoms and bonds undergoing changes (the reaction centre) together with their local environment. The size of this local environment is often defined by a radius parameter, which tunes the level of specificity to reflect enzyme–substrate recognition. By decoupling the reaction mechanism from specific examples, templates support the discovery of new pathways, reaction prediction, and the systematic exploration of alternative biochemical routes.

Template-based strategies are widely used in computer-aided retrosynthesis and retro-biosynthesis tools, alongside template-free machine-learning approaches, both increasingly integrated with deep-learning models [2–4].

**Figure 1.**
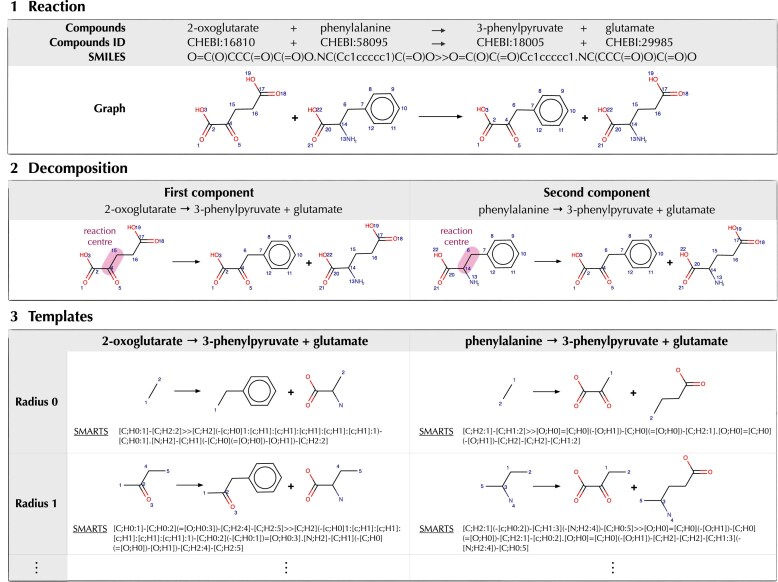
Example of template generation from reaction RHEA:25152. (**1**) The reaction converts 2-oxoglutarate and phenylalanine into 3-phenylpyruvate and glutamate (EC 2.6.1.5, 2.6.1.57); the atom–atom mapping is performed using RXNMapper. (**2**) The mapped reaction is decomposed into its mono-substrate components, with reaction centres highlighted. (**3**) SMARTS templates are generated at increasing radii around each reaction centre. Examples are shown for radii 0 and 1. Only the forward direction is displayed, reverse templates are obtained similarly, by swapping substrates and products in step 1. Chemical structures were drawn using MarvinSketch (ChemAxon, https://chemaxon.com).

Since 2019, RetroRules [5] has provided such templates and, as a unique resource, has offered multi-radius variants, alongside sequence–aware scoring and programmatic access. It has underpinned multiple computational design pipelines [6–9], analytical tools [10–14], and metabolic engineering studies [15–18]. An overview of the template generation process is shown in Fig. 1, using a concrete biochemical example to illustrate the concepts of reaction centre, radius, and reaction templates.

In the years following the original release, both the biochemical curation landscape and cheminformatics tooling have advanced. On the curation side, databases such as MetaNetX [19] and Rhea [20] expanded and refined their coverage, while UniProt [21] continued to consolidate protein evidence and cross–references. On the tooling side, transformer–based atom–atom mapping has improved mapping accuracy and robustness [22, 23]. Meanwhile, expectations grew for user-friendly web interfaces [24, 25], stable APIs, and reproducible distribution formats.

Beyond these technical developments, recent works [26, 27] have emphasized the value of combining biochemical and organic reaction knowledge to design hybrid pathways mixing enzymatic and chemical steps. The present update aims to make RetroRules more accessible, not only as a biochemical database but also as a general framework supporting hybrid pathway design across chemistry domains.

Here, we present a major update of RetroRules that addresses four core objectives:

1. Multi–dataset integration: Templates and reactions from biochemical sources (MetaNetX, Rhea) and a large corpus of organic reactions (USPTO-50k) are integrated. This unification enables extended collections of templates spanning both biochemistry and chemistry domains.
2. Expanded and refined template encoding: The radius range now extends to 0–10 (previously 1–8). Template encoding has been simplified by using implicit hydrogens to align with common cheminformatics toolkits and accelerate its application. Mass-imbalanced reactions are now included, increasing the coverage of enzymatic transformations.
3. Improved mapping and sequence evidence: Atom–atom mapping now relies on RXNMapper [22], improving accuracy over the earlier RDT method [23]. Sequence evidence is updated using a recent UniProt release and summarized as a normalized sequence-support score within the range $[ {0,1} ]$ to facilitate ranking.
4. Usability, performance, and access: A redesigned website now supports multi–criteria filtering (dataset, radius, EC number, reaction ID), deduplicated provenance-aware views, and exports. A reworked REST API mirrors these new capabilities. Previous releases remain available for reproducibility.

## Results

### Data sources and scope

#### Biochemical datasets

Biochemical reactions were updated from MetaNetX [19] (v4.5) and Rhea [20] (release 139), both providing biochemical transformations with cross-references to compounds (e.g. ChEBI [28]) and EC assignments. Original reaction identifiers are retained for traceability. Sequence evidence is updated via Rhea and MetaNetX cross-references to UniProt; accessions are associated with biochemical reactions and propagated to derived templates. EC numbers are dynamically cross-linked to BRENDA [29] entries through the Identifiers.org central registry [30].

#### Chemical dataset

To complement biochemistry, the USPTO-50k [31] corpus was added, a curated subset of reactions extracted from US patents (1976–2016) based on the work of Lowe [32] and extensively used as a benchmark dataset in computational chemistry and machine learning for synthesis planning. It contributes a large and diverse set of organic transformations, thereby extending functional groups and context coverage beyond enzyme-reported reactions. USPTO entries do not contribute sequence accessions.

#### Identity and provenance

Each canonical template is defined by its canonical SMARTS pattern, linked to its source datasets and reactions, substrates and products, and (when available) EC annotations and UniProt accessions. In the web interface, deduplicated views merge identical templates across datasets while preserving dataset-specific evidence and tagging their origin as ‘biochemical’ or ‘organic chemistry’. This scope unites enzyme-catalysed biochemistry with broader organic reactivity while maintaining provenance for downstream analysis.

### Template extraction pipeline

Template generation follows the RetroRules 2019 pipeline: curated reactions are extracted and filtered to remove non-transforming or underspecified entries, the reaction centre is located by atom–atom mapping, multi-substrate reactions are decomposed into mono-substrate components in both directions requiring product atoms to inherit from the focal substrate, optional cofactor pruning is applied, and SMARTS templates are generated at multiple radii around the reaction centre (corresponding to half the ‘diameter’ used in earlier RetroRules versions). This process is illustrated in Fig. 1 on a concrete example (reaction RHEA:25 152), showing the successive steps from atom–atom mapping to the generation of templates at increasing radii. Detailed methods are described in the original RetroRules paper [5] and in the online documentation at https://retrorules.org/docs.

Relative to 2019, explicit-hydrogen syntax is no longer used, reducing the variance in representation and speeding up downstream use [33], and structural normalization now includes stereochemistry stripping prior to template generation. Atom–atom mapping migrated from the MCS-based Reaction Decoder Tool [34] (RDT) to the transformer-based RXNMapper (v0.4.2) [22], with a reported accuracy improvement to 83.74% for RXNMapper, compared to 76.23% for RDT [23]. For biochemical reactions, cofactor pruning uses a curated list from Duigou *et al.* [5] and the Schneider *et al.* [31] list for the USPTO-50k dataset. Both cofactor lists are provided as Supplementary data. Mass-imbalanced reactions are now included, expanding enzymatic coverage. Each candidate template must pass a round-trip quality check: applying the template back to the source substrate must regenerate the expected product(s).

### Template encoding

Templates are generated as SMARTS patterns [1] for radii 0–10 (previously 1–8), enabling both highly general (radius 0) and highly specific (radii 9 and10) contexts. The encoding adopts implicit hydrogens, aligning with the default options of major cheminformatics toolkits and yielding smaller pattern sizes (on average reduced by a factor of 2.3; Supplementary Table S1) and substantially faster pattern applications (from 8- to 200-fold faster; Supplementary Table S2) by reducing graph size and canonicalization overhead. Additionally, the set of atomic primitives is kept minimal: atom symbol, aromatic/aliphatic status, atomic number, and hydrogen count, as well as charges only when required to preserve structural consistency. Combined with canonical SMARTS generation (RDKit-based procedures adapted to SMARTS), this minimizes representation variance and promotes merging of previously near-duplicated patterns into unique canonical forms. Quantitatively, across radii, between 12% and 37% of templates from RetroRules 2019 were consolidated into unique canonical forms in the updated extraction pipeline (Supplementary Table S3). Each template instance retains dataset labels (MetaNetX, Rhea, USPTO) and provenance, while the user interface offers deduplicated views that aggregate evidence across datasets without losing the per-dataset breakdown.

### Handling mass-imbalanced reactions

Upstream biochemical resources (MetaNetX v4.5, Rhea rel. 139) include biologically valid reactions with incomplete or generic stoichiometry (e.g. implicit protons/water, lumped cofactors, class placeholders). The previous release excluded such entries, limiting coverage and discarding transformations with clear mechanistic cores. The 2026 update now admits mass-imbalanced biochemical reactions when atom–atom mapping could be successfully carried out. This increases coverage of biochemically relevant reactions and contributes to the expansion of level-4 EC coverage (Table 1). Quantitatively, inclusion of mass-imbalanced reactions from MetaNetX and Rhea increased the number of distinct templates by 9% and expanded the EC coverage by 1.5% (Supplementary Table S4).

**Table 1.** Content of RetroRules from 2019 to 2026

	Dataset	Release		Evolution
		2019	2026	
Source reactions	MetaNetX	16 786	31 076	×1.8
	Rhea	–	11 624	–
	USPTO	–	49 998	–
	Total	16 789	92 698	×5.5
Templates	MetaNetX	168 441	302 879	×1.8
	Rhea	–	125 842	–
	USPTO	–	814 990	–
	Total	168 441	1 174 216	×7.0
Templates with ≥1 UniProt	Total	134 599	143 414	×1.1
EC level–1	Total	6	7	
EC level–2	Total	60	72	×1.2
EC level–3	Total	219	250	×1.1
EC level–4	Total	4374	5796	×1.3

The numbers report the counts per dataset (MetaNetX, Rhea, USPTO), the total corresponding to the union of all datasets, the number of templates annotated with at least one UniProt sequence, and the EC class coverage from the top to the fourth level. A dash indicates that the dataset was not included in the 2019 release.

### Release content and coverage

#### Global volumes and per-dataset counts

The 2026 release contains 1 174 216 canonical templates derived from 92 698 source reactions: 31 076 from MetaNetX, 11 624 from Rhea, and 49 998 from USPTO. By dataset, the templates total 302 879 (MetaNetX), 125 842 (Rhea), and 814 990 (USPTO). Compared with 2019 (168 441 templates, 16 789 source reactions), this represents a nearly seven-fold expansion, driven by the inclusion of USPTO and broader coverage in Rhea and MetaNetX. Table 1 provides a detailed summary of counts.

#### Dataset overlaps

**Figure 2.**
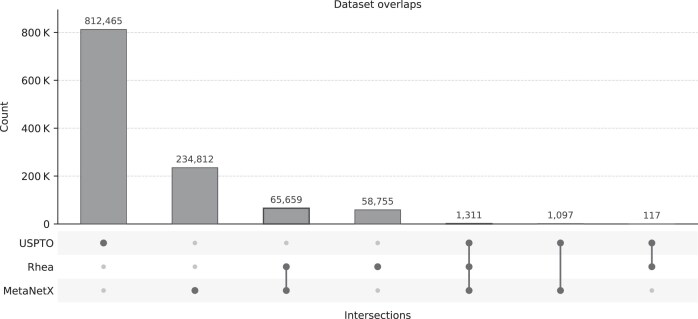
Upset plot of overlaps between MetaNetX, Rhea, and USPTO. Top bars show the size of each intersection. The bottom dot–line matrix encodes set membership.

An overlap analysis is shown in Fig. 2. The sizable MetaNetX–Rhea intersection (65 659 templates) reflects a convergent biochemical curation, which is expected considering MetaNetX proposes a reconciliation of chemical namespaces between multiple biochemical databases including Rhea, whereas overlap with USPTO remains minimal, underscoring the domain differences (∼2500 templates). The datasets therefore act complementarily, with USPTO adding organic-chemistry patterns that extend the biochemical space.

#### Template distributions across radii

**Figure 3.**
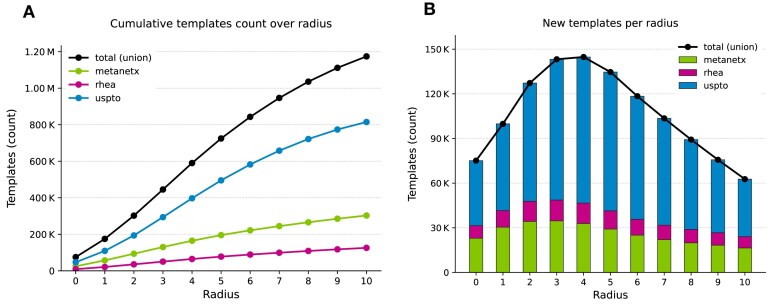
Count of unique templates across radii and datasets. (**A**) Cumulative unique templates count across increasing radii. (**B**) Incremental contribution of each radius to the total unique template count. Stacked bars show the number of distinct templates first generated at the indicated radius within each dataset.

Templates are encoded at radii 0–10 around the reacting atoms. As shown in Fig. 3A, the cumulative number of unique templates increases with radius, reflecting the broader chemical context captured around each reaction centre. Growth is more pronounced at smaller radii (0–3) and gradually tapers at larger ones as the local environment saturates (i.e. once the full structure of the reacting substrate is encompassed, extending the radius no longer introduces novel atomic configurations). Figure 3B shows the incremental contribution of each radius, highlighting that most new templates are generated between radii 2 and 5, after which the number of newly generated patterns gradually decreases.

#### EC coverage

Across biochemical sources (MetaNetX, Rhea), the release covers 250 third-level and 5796 fourth-level EC classes, which represent 1.1-fold and 1.3-fold increases compared with the 2019 release (Table 1; 2019: 219 level-3 ECs; 4374 level-4 ECs). Table 1 shows partitions across the top-level EC classes. Inclusion of well-mapped, mass-imbalanced reactions contributes to the broader coverage of the biochemical reaction space (Supplementary Table S4).

### Sequence evidence and scoring

Sequence annotations derive from biochemical sources (Rhea/MetaNetX), while USPTO contributes none. The number of templates with at least one UniProt accession slightly increases from 2019 (134 599) to the 2026 release (143 414), as shown in Table 1. To provide a simplified ranking key, the previous sequence evidence score [5] is normalized such that $s\in [ {0,\ 1} ]$. The score, defined as $s = 1/\sqrt[r]{n}$, where $n$ is the number of enzyme sequence clusters supporting the template and $r$ is a regularization parameter, ensures comparability across datasets and radii by construction (see Supplementary Note S5). Higher $s$ values therefore indicate less ambiguous sequence support. The score is exposed in the UI and API, enables sequence-aware prioritization in pathway searches, and readily combines with other criteria (e.g. chemical similarity or EC agreement) without retuning. Additionally, templates derived from spontaneous reactions are no longer penalized for missing sequence evidence; they receive the best score of 1 rather than being downweighed.

### Web application and online template generator

**Figure 4.**
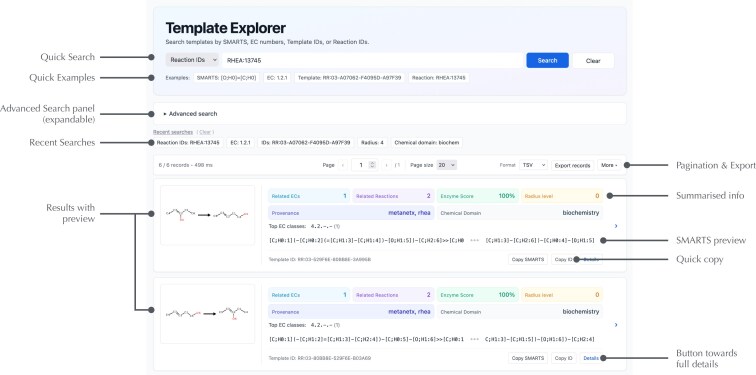
Template Explorer preview. From top to bottom: the Quick Search allows single-filter search, example queries enable users to test searches easily, while the Advanced Search panel gives access to all combinations of search settings and sorting. Template records appear as cards with visual previews, summary chips (related ECs and reactions, sequence-based score, radius, dataset provenance, chemical domains), inline SMARTS preview, one-click copy, and a link to the detailed view.

The website has been fully redesigned for navigation, search, and template export, in addition to a responsive layout for desktop, tablet, and mobile use. Template Explorer now supports multi-criteria search across datasets (MetaNetX, Rhea, USPTO), radius values (0–10), EC number (including partial levels such as ‘2.7.1’), and reaction ID, with customizable sorting. Results are deduplicated based on canonical SMARTS and displayed as compact cards with summary chips for key attributes such as radius range, EC count, related reaction count, sequence support, and data source provenance. Each hit renders the SMARTS pattern and provides quick actions to copy the SMARTS and template ID or open a permalink. The annotated screenshot in Fig. 4 illustrates the new layout and result summaries.

The Template Detail View aggregates evidence across datasets while preserving per-dataset sources and links. Pages list source reactions, radius coverage, EC assignments (with cross-links to BRENDA [29]), linked compounds (with link-outs to MetaNetX and ChEBI), and associated UniProt accessions when available. Original reaction, substrate, and product identifiers are retained, and omitted cofactors are disclosed.

The Online Template Generator was rebuilt to implement the updated extraction and encoding workflow. From a user-provided reaction (SMILES), the tool performs transformer-based atom–atom mapping, mono-substrate decomposition, implicit-hydrogen encoding, and emits templates at radii 0–10, including cases derived from mass-imbalanced reactions when a coherent reaction centre exists. Generated templates appear in the same UI as the Explorer for immediate inspection, filtering, and export (TSV/CSV/JSON), ensuring that interactive exploration and on-the-fly generation share one consistent representation.

### Programmatic access

The REST API has been redesigned to mirror the Template Explorer’s multi-criteria search: filters for dataset, EC (exact or prefix such as ‘2.7.1’), radius (0–10), reaction IDs, and template IDs. Results are returned as typed JSON, deduplicated by canonical SMARTS, with provenance counts and sequence-support scores when available. Documentation follows the OpenAPI specification and is published online with interactive forms, enabling direct, in-browser query testing. Pagination and soft rate limits protect the service (default page size 100), while larger datasets are provided via the Downloads page. For high-throughput workflows, prebuilt per-dataset bundles mirror API fields to ensure bit-identical results with the online service.

### Interoperability and integrations

RetroRules provides reaction (MetaNetX and Rhea), compound (MetaNetX and ChEBI), EC number (BRENDA), and protein (UniProt) identifiers for external registries. The dataset provenance remains attached to templates.

Template identifiers adopt a deterministic, three-layer design: RR:AAAAAA-BBBBBB-CCCCCC, where A hashes the substrate subgraph (heavy-atom connectivity), B hashes the product subgraph, and C hashes the canonical SMARTS of the template. The scheme, inspired by InChIKey layering, enables fast, MCS-free grouping and retrieval—e.g., selecting all templates that share the same substrate motif via the A segment, or clustering alternative product contexts via B—while C identifies the complete mapped transformation. All template identifiers were verified to be unique across the entire release; no collisions were found.

Integration into workflows is provided via a RetroRules Galaxy node, which parameterizes queries (dataset, radius, EC-number filters, reaction, or template IDs) and returns TSV/CSV/JSON suitable for batch pipelines. This node lowers the entry barrier for non-specialists while preserving full provenance in outputs, supporting routine pathway design and rule selection, and enabling end-to-end, reproducible pipelines through Galaxy-based environments [35].

## Discussion

This update integrates consolidated biochemical resources (MetaNetX v4.5, Rhea release 139) with broader organic chemistry (USPTO-50k), expanding the template space by ∼7× and EC coverage by ∼1.3×. Extending encoding to radii 0–10 provides fine-grained control of specificity, with radius 0 capturing the reaction centre and higher radii enforcing context, which also supports modelling of enzymatic promiscuity. The shift to implicit-hydrogen encoding and minimal atomic primitives makes SMARTS more compact and faster to apply (∼5–10× in practice), lowering computational costs for pathway search. Migration from an MCS-based approach to RXNMapper improves mapping accuracy, supporting more reliable decomposition and template generation. The website redesign improves accessibility with faceted search by dataset, radius, EC numbers, and reaction IDs, provenance-aware deduplication, sortable results, and per-template pages with exports and link-outs. The REST API mirrors these capabilities with typed filters, and an OpenAPI guide enabling example executable queries.

Crucially for (bio-)retrosynthesis, multi-radius templates enable control of the branching factor and route plausibility in backward search. Lower radii broaden exploration and increase coverage of candidate disconnections, while higher radii constrain context to reduce false positives. Sequence evidence and EC constraints supply priors for ranking, and faster matching allows deeper or broader searches under fixed time constraints. In this context, a reaction template database serves as an accessible entry point for scalable retrosynthesis and pathway design, offering consistent abstractions with controllable specificity, fast matching, and programmatic access for ranking, pruning, and integration into automated workflows.

Patent-driven chemistry and nature’s metabolism occupy largely different neighbourhoods of reaction space. This is illustrated by the tiny overlap of USPTO templates with the biochemical sets (2525 shared templates, which represent ∼0.2% of USPTO templates), indicating complementarity rather than redundancy. This breadth helps connect biochemical reactivity to alternative contexts when needed in modelling, without being a prerequisite for the core biochemical use cases.

Two trade-offs deserve attention. First, templates from reactions not fully mass-balanced are included. Mass-imbalanced biochemical reactions can arise from missing compounds and result in inconsistent atom mappings, making them slightly less reliable, yet they may capture valid conversions not otherwise represented. Retaining them without artificial rebalancing preserves transparency while modestly broadening biochemical coverage (∼1.5% increase in EC coverage, Supplementary Table S4). Second, stereochemistry is dropped relative to 2019. Stereochemistry in SMARTS is brittle across toolkits and sources, and consistent handling requires precise local context that is not uniformly annotated. Tools such as RDChiral [36] provide improved consistency on the template application side, yet heterogeneous encodings and incomplete stereochemical annotation in metabolic resources remain uneven, limiting reliable propagation at scale.

The integration of enzymatic and synthetic chemistry has emerged as a major objective in computer-aided synthesis planning (CASP). Finnigan *et al.* demonstrated this integration with RetroBioCat [37], which formalizes expert knowledge on biocatalytic cascades, while Levin *et al.* extended this concept by coupling enzymatic and synthetic neural models to automatically design hybrid retrosynthetic routes [26]. In this context, RetroRules 2026 provides a complementary and enabling foundation for such developments by systematically harmonizing biochemical and organic reaction data within a unified template representation. This cross-domain standardization facilitates algorithmic interoperability between metabolic and synthetic CASP tools, enabling hybrid searches and reuse of knowledge across domains, and thereby directly supporting the automated design of pathways that combine enzymatic and chemical steps.

Priorities for the next cycle include multi-criteria scoring (combining sequence support, structural context, and observation frequency), thermodynamic evaluation of reactions to detect unrealistic energy profiles and improve pathway plausibility, and broader handling of cofactors and protonation states to enhance comparability between datasets. These evaluations will help flag less plausible transformations and prevent biologically unrealistic pathways, thereby strengthening the reliability of pathway design and ranking. Together with stable identifiers, open formats, and programmatic access, these developments aim to keep RetroRules a FAIR and transparent resource for large-scale pathway design, reaction prediction, and enzyme engineering.

## Conclusions

RetroRules 2026 delivers a unified, multi-dataset template resource that couples curated biochemistry (MetaNetX v4.5, Rhea rel. 139) with diverse organic reactivity (USPTO-50k) under explicit provenance. Extended radii (0–10) provide tuneable specificity from centre-only to highly contextual patterns, supporting enzymatic promiscuity modelling, while implicit-hydrogen encoding and minimal atom primitives compress rules and accelerate application. Atom–atom mapping upgraded to RXNMapper improves accuracy, and sequence annotations are refreshed and normalized for ranking. The inclusion of reactions that are not fully mass-balanced expands biochemical coverage without constraining stoichiometry-sensitive analyses. A redesigned website, an overhauled Online Template Generator, and an OpenAPI-typed REST API expose faceted search, deduplicated provenance-aware views, stable identifiers, and convenient exports suited to automated pipelines. Versioned, DOI-backed bundles and accessible archives preserve reproducibility and long-term interoperability. Together, these advances provide a practical substrate for retrosynthesis, retro-biosynthesis, pathway discovery, and reaction prediction at scale, with next steps aimed at multi-criteria scoring, thermodynamic and stoichiometric quality signals, and generalized cofactor handling.

## Supplementary Material

gkaf1261_Supplemental_Files

## Data Availability

RetroRules is accessible at https://retrorules.org, which provides the Template Explorer, Online Template Generator, and a documented REST API (https://retrorules.org/apidocs, OpenAPI). Dataset-specific bundles (MetaNetX, Rhea, USPTO) may be downloaded from the dedicated page (https://retrorules.org/download) in TSV, CSV, and JSON formats. The current release is 3.0.0; previous versions (rr01–rr02) remain available. Data are distributed under the CC BY 4.0 license, with upstream licenses applying. All releases have persistent DOIs, which are listed on the website.
