# Supplementary material for: RetroRules 2026: an expanded database combining biochemical and organic reaction templates for pathway discovery
Source: Nucleic Acids Res. 2025 Dec 8;54(D1):D1799–806. doi: 10.1093/nar/gkaf1261 (PMC12807659; doi:10.1093/nar/gkaf1261)
Supplement: gkaf1261_Supplemental_Files [file gkaf1261_supplemental_files.zip › Supplementary_S1-S5.pdf]

## Supporting information

# RetroRules 2026: an expanded database combining biochemical and organic reaction templates for pathway discovery

Thomas Duigou[1,*], Philippe Meyer[1], Jean-Loup Faulon[1,2]

1: Université Paris-Saclay, AgroParisTech, INRAE, Micalis Institute, 78350, Jouy-en-Josas, France
2: The University of Manchester, Manchester Institute of Biotechnology, Manchester M1 7DN, U.K
*: corresponding author

**Table S1. Reduction in template size between RetroRules 2019 and 2026 encodings.**

The 2019 encoding corresponds to the RetroRules 2019 release built from MetaNetX v3.1. The 2026 encoding represents templates re-generated from the same dataset using the updated RetroRules SMARTS format, isolating the effect of the new encoding from database changes.

Template size is measured as the number of characters in the SMARTS string. For each reaction radius, mean lengths are reported together with the relative change [$\% = (len_{2026} - len_{2019})\ /\ len_{2019}$] and the multiplicative reduction coefficient.

| Radius | Mean template length (chars) | | Δ mean length (%) | Coefficient reduction (x) |
|---|---|---|---|---|
| | 2019-encoding | 2026-encoding | | |
| 1 | 482 | 218 | -54.8 | 2.2 |
| 2 | 579 | 258 | -55.4 | 2.2 |
| 3 | 680 | 299 | -56.0 | 2.3 |
| 4 | 774 | 336 | -56.6 | 2.3 |
| 5 | 852 | 366 | -57.0 | 2.3 |
| 6 | 915 | 390 | -57.4 | 2.3 |
| 7 | 967 | 411 | -57.5 | 2.4 |
| 8 | 1016 | 430 | -57.7 | 2.4 |
| Average | 783 | 338 | -56.8 | 2.3 |

**Table S2. Reduction in template application time between RetroRules 2019 and 2026 pattern encodings.**

The 2019 encoding corresponds to the RetroRules 2019 release generated from MetaNetX v3.1. The 2026 encoding refers to templates re-generated from the same dataset using the updated RetroRules SMARTS format, isolating the effect of the new encoding from changes in source databases.

Execution time measures the application of each reaction template to its source reactant. For each reaction radius, mean times are reported along with absolute savings and multiplicative speedup (2019 / 2026 encoding).

| Radius | Mean execution time (ms) | | Mean saving (ms) | Speedup (x) |
|---|---|---|---|---|
| | 2019-encoding | 2026-encoding | | |
| 1 | 1.090 | 0.140 | 0.950 | 8 |
| 2 | 2.130 | 0.061 | 2.069 | 35 |
| 3 | 3.205 | 0.052 | 3.153 | 62 |
| 4 | 5.457 | 0.054 | 5.403 | 101 |
| 5 | 8.599 | 0.058 | 8.541 | 147 |
| 6 | 11.664 | 0.063 | 11.602 | 186 |
| 7 | 15.397 | 0.072 | 15.325 | 215 |
| 8 | 18.559 | 0.073 | 18.486 | 255 |

**Table S3. Template consolidation across reaction radii.**

For each reaction radius, the table reports the number of distinct templates from RetroRules 2019 (RR-02), the subset of merged templates within RR-02, and the resulting number of canonical templates produced through the RetroRules 2026 generation pipeline. The merged fraction (%) indicates the proportion of RR-02 templates unified under a single canonical SMARTS representation.

| Radius | Distinct templates (RR-02) | Merged templates (RR-02) | New canonical templates | Merged fraction (%) |
|---|---|---|---|---|
| 1 | 15,505 | 5,799 | 2,412 | 37.4 |
| 2 | 20,380 | 5,459 | 2,669 | 26.8 |
| 3 | 23,385 | 4,712 | 2,554 | 20.1 |
| 4 | 24,750 | 4,162 | 2,382 | 16.8 |
| 5 | 25,530 | 3,688 | 2,185 | 14.4 |
| 6 | 26,050 | 3,521 | 2,119 | 13.5 |
| 7 | 26,432 | 3,358 | 2,074 | 12.7 |
| 8 | 26,711 | 3,258 | 2,064 | 12.2 |

**Table S4. Contribution of Rhea and mass-imbalanced reactions to biochemical template and EC coverage**. Starting from a MetaNetX-only baseline (v4.5), the table reports the numbers of distinct templates and level-4 EC numbers before inclusion, and the additional elements gained after admitting mass-imbalanced reactions and/or integrating Rhea reactions.

| Scenario | Data Type | Baseline | Added | Increase (%) |
|---|---|---|---|---|
| Include Rhea | Template | 278,538 | 59,058 | 21.2 |
| | EC number | 5,440 | 277 | 5.1 |
| Include imbalanced (MNX) | Template | 278,538 | 24,341 | 8.7 |
| | EC number | 5,440 | 80 | 1.5 |
| Include Rhea + imbalanced (MNX + Rhea) | Template | 278,538 | 83,213 | 29.9 |
| | EC number | 5,440 | 356 | 6.5 |

**Note S5. Definition of the template score**

Each reaction template in RetroRules is associated with a sequence support score that estimates its degree of biochemical uncertainty. This metric quantifies how specifically a rule can be linked to enzyme sequence evidence derived from biochemical databases.

*Definition*

$$score = \frac{1}{\sqrt[r]{n}}$$

where:

- $n$ is the number of distinct, non-redundant sequence clusters that are associated to the source reactions modelled by the template. Sequence clusters are computed from UniProt accessions using CD-HIT.
- $r$ acts as regularization parameter and is set to $1/4$

*Interpretation*

- High scores (close to 1) correspond to templates associated to a single enzyme cluster, indicating low biochemical uncertainty.
- Lower scores correspond to rules supported by multiple unrelated enzyme clusters, reflecting higher uncertainty in the biochemical specificity of the transformation.
- The index of the root $r = 4$ regularizes the score behaviour, ensuring that score differences remain moderate even when rules are supported by large numbers of sequences, while preserving the overall rank order.
